# Identification of genetic determinants that promote biofilm growth under heterotrophic conditions in *Cupriavidus necator* using transposon enrichment

**DOI:** 10.1016/j.bioflm.2026.100395

**Published:** 2026-09-01

**Authors:** Janek René Weiler, Christian Jonas Lapp, Johannes Gescher, Miriam Edel

**Affiliations:** Institute of Technical Microbiology, Hamburg University of Technology, Kasernenstraße 12, Hamburg, 21073, Germany

**Keywords:** *Cupriavidus necator* H16, *Ralstonia eutropha* H16, Biofilm formation, Transposon insertion library, Transposon screen, EAL-domain, Cyclic di-GMP

## Abstract

*Cupriavidus necator* is a metabolically versatile β-proteobacterium of growing interest for auto- and heterotrophic bioprocesses, yet the genetic determinants governing its biofilm formation remain largely uncharacterized, particularly under process-relevant heterotrophic conditions. Here, we applied a forward-genetics transposon-enrichment approach to identify loci which promote surface-associated growth. A high-density mini-Tn*5* mutant library (26,185 insertion clones, exceeding the >17,000 required for genome-wide coverage) was cultivated as a biofilm in a microfluidic flow-cell system on fructose for 168 h, and the surface-associated community was characterized by deep sequencing. Twelve genes showed significantly elevated insertion frequencies, several with documented links to biofilm formation in other bacteria, including the ferrous-iron uptake system (*feoA*/*feoB*), *galU*, and a GSDEF/EAL dual-domain protein. The gene B2043 (E6A55_RS29530), encoding this c-di-GMP-metabolizing protein, was selected for validation by markerless deletion. Under static conditions, the ΔB2043 mutant showed a 1.69 ± 0.06-fold increase in biofilm-associated biomass (p = 5.16 × 10^−15^). Under flow-through conditions, the mutant attached faster, entered exponential growth ∼10 h earlier, reached its biovolume plateau ∼16 h earlier than the wild-type, and formed distinct tower-like structures. These results identify B2043 as a negative regulator of biofilm formation acting predominantly during attachment, provide the first experimental evidence for c-di-GMP-dependent biofilm regulation in *C. necator* H16, and establish a functional-genomics framework — together with eleven further candidate loci — for engineering productive biofilms in this organism.

## Highlights

• Tn5 screen identified 12 candidate genes that promote biofilm growth in *C. necator.*
• The GGDEF/EAL-domain gene B2043 was selected for functional validation.
• Deletion of B2043 increased static biofilm biomass by 1.69-fold.
• The mutant attached faster, and formed tower-like structures.
• First experimental evidence for c-di-GMP-dependent biofilm regulation in *C. necator.*

## 1 Introduction

*Cupriavidus necator* is a metabolically versatile, Gram-negative β-proteobacterium that can thrive on a wide range of organic substrates using oxygen or nitrate as electron acceptors. Moreover, it can grow as a lithoautotroph using hydrogen as electron and energy source. Its capacity to accumulate value-added products such as polyhydroxyalkanoates together with its genetic tractability are traits that are harnessed for applied auto- and heterotrophic bioprocesses [[1], [2], [3]]. However, there is still a lack of knowledge regarding fundamental regulatory determinants in the cell. For instance, only limited knowledge exists on the genetic determinants that govern biofilm development in *C. necator*, particularly under process-relevant conditions with industrially relevant carbon sources.

However, productive surface-associated growth is often desirable, as biofilm-based processes can enable higher cell densities, improve robustness against environmental fluctuations, and enable continuous operation compared to planktonic cultures as well as higher productivities in biotechnological applications [4]. Biofilms also facilitate the formation of gradients of nutrients, metabolites, and signaling molecules that enhance stress tolerance and productivity, making surface-associated growth a potentially attractive option for large-scale biomanufacturing [2,5]. At the same time, biofilm formation is a complex, multigenic trait involving coordinated regulation of adhesion, extracellular matrix production, quorum sensing, and metabolic shifts necessitating deeper knowledge to optimize biofilm formation for industrial applications [6].

Forward genetic approaches, based on random insertional mutagenesis, offer a powerful route to explore the genetic landscape underlying complex traits such as biofilm formation, in a largely unbiased manner [7]. By establishing a correlation of random gene disruptions to phenotypic outcomes, these methods enable genome-wide identification of determinants that contribute to cellular fitness and adaptation [8,9]. In particular, dense transposon mutant libraries can be used in pooled selection experiments, where competition between thousands of mutants under defined conditions reveals gene disruptions that enhance or impair fitness under certain process conditions. Transposon insertion studies are among the most widely used methods for conducting functional genetic screens in bacteria at a genome-wide scale. The random integration of transposons allows for the screening of nearly all possible beneficial gene disruptions in selection experiments with minimal effort.

When combined with high-throughput sequencing (Tn-seq), such screens enable genome-wide mapping of loci that contribute to surface attachment, extracellular matrix production, extracellular DNA release, or the transition between planktonic and biofilm growth. While transposon insertion sequencing has been widely applied to study antibiotic resistance, and stress tolerance in many bacteria, which also led to the identification of *Pseudomonas* and *Bacillus* biofilm regulators, its use to dissect and improve biofilm formation in *C. necator* under process-relevant heterotrophic conditions remains largely unexplored [[10], [11], [12], [13], [14]].

Recent studies have demonstrated the power of transposon screens to uncover biofilm regulators across bacterial taxa. For example, Tn-seq in *Escherichia coli* identified a cyclic di-GMP (c-d-GMP) hydrolase mutant strongly enriched through matrix hyperproduction during biofilm formation [10]. Similarly, *Pseudomonas aeruginosa* screens revealed BosR, a one-component regulator which seems to have a conserved role in *P. aeruginosa* biofilm growth [15]. Tn-seq in *Bacillus subtilis* further identified *sinR* repressor mutants exhibiting hyper-biofilm phenotypes through SinR-mediated activation of matrix genes [16]. These examples illustrate how transposon mutagenesis preferentially leads to the identification of higher-order regulators rather than individual structural genes, providing systems-level insights into biofilm circuit architecture. When applied to *C. necator*, this approach has the potential to identify both known conserved regulators and novel species-specific factors that coordinate heterotrophic biofilm development, as well as disrupted genes that contribute to a general fitness benefit under the experimental conditions.

In this study, we set out to systematically identify genetic factors that enhance biofilm formation of *C. necator* during heterotrophic biofilm growth and to directly exploit these findings for strain improvement. To this end, we generated a high-density transposon mutant library in which each cell carries a single insertion in a random gene. Following a week-long biofilm cultivation period, during which the biofilm reached maturity, the composition of the resulting cell community of the transposon library was analyzed by deep sequencing. This analysis was conducted to determine which insertions had become significantly enriched in the surface-associated community, thereby pinpointing genes whose disruption confers a competitive advantage for the biofilm lifestyle. One of the most frequent and reproducibly enriched mutations, identified across biological replicates, was then reconstructed in an otherwise wild-type background using marker-less genetic modification, decoupling its biofilm-promoting effect from potential interactions within the mixed-library context. The resulting engineered strain was subsequently characterized in detail for its biofilm formation capacity and architecture relative to the parental wild-type strain under flow conditions. Thus, this work establishes a functional genomics framework for uncovering and validating biofilm-promoting mutations in *C. necator* and identifies customizable strains for enhanced surface-associated growth in heterotrophic bioprocesses.

## 2 Material and methods

### 2.1 Strains and cultivation

All strains used in this study are listed in Table 1.

**Table 1:** Strains used in this study.

Strain	Strain number	Relevant Genotype	Source
*E. coli* WM 3064	***JG 98***	*thrB1004 pro thi rpsL hsdS lacZ* Δ*M15RP4–1360* Δ(*araBAD*)567 Δ*dapA1341*::[*erm pir*(*wt*)]	W. Metcalf, University of Illinois
*C. necator* H16	***JG 1070***		*DSM 428;* [17,18]
*C. necator* H16	***JG 2152***	Δ E6A55_RS29530	*This study*

*C. necator* strains were grown as pre-cultures in lysogeny broth (LB) at 30 °C with shaking at 180 rpm. For the biofilm growth, the strains were cultured in minimal media (MM; DSMZ medium 81, modified with 2.5 mg of ferric ammonium citrate instead of the standard 50 mg) at 30 °C. Heterotrophic growth was achieved by supplementing with 20 mM fructose. *E. coli* WM3064 was cultivated in LB medium at 37 °C with 0.3 mM diaminopimelic acid (DAP) supplementation.

Kanamycin was added, when necessary, at a concentration of 250 μg/ml for *C. necator* and 50 μg/ml for *E. coli*. Ampicillin was used at 100 μg/ml instead of kanamycin for the stabilization of pBAMD1-2 in *E. coli*.

Solid media were prepared by adding 2% agar to the respective liquid medium. All media and stock solutions were sterilized by autoclaving at 121 °C for 20 min. Heat-sensitive components (antibiotics, vitamins, and fructose) were sterilized separately by filtration through a 0.22 μm filter (Sarstedt AG & Co., Nümbrecht, Germany).

Cryopreservation was used to store bacterial strains long term. To accomplish this, 1 mL of a densely colonized liquid culture was mixed with 500 μl of glycerol, and the mixture was frozen in liquid nitrogen. The mixture was then stored at −80 °C. For experiments, the strains were plated on LB-Agar-plates (2%), which were stored at 4 °C and used for up to a week to inoculate precultures.

### 2.2 Creation of the mutation insertion-library

To identify genes involved in heterotrophic biofilm formation in *C. necator*, a transposon-induced mutation library was created. To this end, the pBAMD1-2 plasmid, carrying a mini Tn5-delivery system was used (Addgene plasmid #61564; https://n2t.net/addgene:61564; RRID:Addgene_61564; [19]). For subsequent deep-sequencing, an I-Scel-recognition site was inserted between the mosaic-ends as the sole change. Insertion of the I-Scel-site, library preparation and subsequent deep-sequencing, and detailed analysis were performed as described in Lapp et al. (2026) [20].

The resulting plasmid was introduced into the diaminopimelic acid (DAP)-auxotrophic *E. coli* strain WM3064 by electroporation. Colony-forming transformants were verified by Sanger sequencing at Eurofins Genomics (Ebersberg, Germany) using the Mix2Seq Kit, and the obtained sequences were compared with the in-silico constructs using Benchling molecular biology tools (San Francisco, USA). A verified strain carrying the correct plasmid was subsequently used as an intermediate donor for conjugation with the *C. necator* wild-type strain. To estimate the minimum number of independent transposon insertions required to achieve a 90% probability of disrupting an average-sized, non-essential gene, the relationship between the number of transformants (n) and the insertion probability (P = ) 0.90 was calculated. The calculation was based on a genome size of (G = ) 7400 kb and an average gene length of (x = ) 1 kb for *C. necator*, assuming random and independent insertion events, according to Krysan et al. (1999) [21] and Clarke and Carbon (1976) [22] as shown in formula 1.

(1) $$Probability\;of\;integration\;P=1-{\left(1-\frac{x\;\left[kb\right]}{G\;\left[kb\right]}\right)}^{n}$$

$$0.9=1-{\left(1-\frac{1\;}{7.400}\right)}^{n}$$

n=17,038

To obtain the required >17,000 transformants, serial dilutions of both strains at an OD_600_ of 1 were plated on LB agar to determine the number of colony-forming cells. The transformed *E. coli* and the target *C. necator* strain were then cultivated separately in liquid LB medium to an OD_600_ of 0.6. Based on the colony counts, at least 1000 colony-forming cells of each strain were combined on 20 LB agar plates each, supplemented with 0.3 mM DAP and incubated at 30 °C for 2 days. This setup was used to limit repeated cell-to-cell contacts. Colony counting confirmed that the minimum number of cells from both strains had been used. The transformed cells were then recovered by washing the plates with fresh LB medium and re-plated onto 20 LB agar plates containing 250 μg/mL Kanamycin for mutation stabilization. After incubation, colonies were counted to verify that the required minimum of 17,000 insertion events had been reached; in total, 26,185 colonies were obtained. Finally, the plates were washed with LB medium containing 250 μg/mL Kanamycin, and the collected cells were stored at −80 °C for deep sequencing and subsequent experiments.

### 2.3 Biofilm cultivation of the mutation library

*C. necator* biofilm cultivation was carried out using a well-established microfluidic system originally described by Hansen et al. (2019) [23]. This system is characterized by the continuous delivery of fresh medium within a confined volume, which facilitates rapid analytical assessment. The microfluidic flow cells were constructed using polydimethylsiloxane (PDMS) and featured a linear embedded channel measuring 54 x 3 × 2 mm (length x width x height) with a total volume of 354 μL. These flow cells were produced using the Sylgard® 184 Silicone Elastomer Kit from Dow Corning (Michigan, USA). The PDMS material was cast into a brass negative mold and then cured at 60 °C for 2 h. Placeholder cannulas were positioned during the curing stage to create three outlets for media inflow, outflow, and organism inoculation. Oxygen plasma treatment (SmartPlasma 10 Pro, Plasma Technology, Herrenberg, Germany) enabled the silicone body to covalently bond to 1-mm-thick cover glass. After bonding, permeable cannulas (0.8 mm diameter) were inserted into the pre-formed cavities to make the channels accessible. These cannulas were then connected to a silicone tubing network with a 1.5 mm inner diameter (Carl Roth, Karlsruhe, Germany). Peristaltic pumps (Reglo ICC model from the ISMATEC product line by Cole-Parmer in Wertheim, Germany) maintained consistent medium delivery to the culture channel. The component assembly employed Luer connectors, and three-way valves facilitated the alternation between inoculation and media supply modes. The system's inlet and outlet both featured collection vessels for medium handling and disposal. Pressure equilibration was achieved by attaching cannulas with sterile filters (0.2 μm; Sarstedt AG & Co., Nümbrecht, Germany) to these vessels. All system components underwent sterilization through autoclaving at 121 °C with 1 bar overpressure for 20 min prior to assembly. Final assembly took place within a sterile environment using a Thermo Scientific MSC Advantage Safety Workbench (Fisher Scientific, Schwerte, Germany). Biofilm optical analysis used optical coherence tomography (OCT) to quantify biofilm volume and height during growth by utilizing the PDMS transparency.

Cultivation within this system took place at 30 °C, at a medium flow rate of 4 ml/h, with MM 81. Inoculation of the system was carried out over 2 h on the side port of the system at 30 °C and with a flow rate of 1 ml/h, while medium flow rate was reduced to 3 ml/h. Optical density of the pre-culture was measured at 600 nm wavelength and adjusted to 0.2 prior to inoculation. To ensure biofilm formation was the only selection-pressure present, the 2 ml of the mutation-library were directly inoculated into the biofilm-cultivating system without preculturing the cells. Experiments were carried out in quadruplicates for the experiment with the mutation-library, or in triplicates for the comparison experiment of the marker-less deletion experiment. Biovolume and growth were measured at the middle section of the cultivation channel via OCT as described previously by Klein et al. (2024) [24].

### 2.4 Marker-less deletion

Marker-less genomic deletion of target genes identified through mutation-library screening in *C. necator* was performed using the suicide vector pMQ150 as described by Windhorst and Gescher [25,26]. The vector was linearized through digestion with BamHI and SalI at 37 °C for 2 h using CutSmart buffer (New England Biolabs, Ipswich, USA). PCR amplification of 500 bp fragments flanking the upstream and downstream regions of the target gene was carried out using primers 1 - 4 (Table 2). Successful gene deletion was confirmed through diagnostic PCR using primers 5 and 6 (Table 2), followed by Sanger sequencing verification (Eurofins Genomics, Ebersberg, Germany, Mix2Seq Kit). Sequence analysis was performed by comparison with in-silico reference sequences using Benchling's molecular biology software suite (San Francisco, USA).

**Table 2:** Primers used in this study.

Target	Primer number	Sequence (5’ - 3′)	Source
*500 bp UP-fragment of B2043*	***for.: 4786***	GTCACGACGTTGTAAAACGACGGCCAGTGCCAAGCTTGCATGCCTGCAGGGTCAGCGTGGCAATGCG	*This study*
	***rev.:4787***	TGCTAAATTGCTAGCGGGTAACAATGTCGACAATTACAGATGGGGCAGGGCTTCCGCCGCCGTTTT	
*500 bp DOWN-fragment of B2043*	***for.: 4788***	AGGTGGAAACAACTTCCCTGACAGGGCGGGGGGCAAAACGGCGGCGGAAGCCCTGCCCCATCTGTAATTG	*This study*
	***rev.: 4789***	AGGAAACAGCTATGACATGATTACGAATTCGAGCTCGGTACCCGGGGATCTTGATGGTCTGCCACGC	
*Test primers (deletion of B2043)*	***for.: 4791***	TCAAGCCAAAGCCGGTCGC	*This study*
	***rev.: 4792***	TCGCGCGTGATCGAGGTCTC	

### 2.5 Analyses

#### 2.5.1 Optical coherence tomography (OCT)

OCT images were taken to non-invasively analyze the growth and three-dimensional structure of biofilms within the cultivation channels. OCT is an imaging technique that uses light scattering, reflection, and interference to produce two- and three-dimensional images in the micrometer range. The device emits a light beam with a short coherence length in the infrared range (930 nm, 100 nm bandwidth) from a source. This beam is split into a reference beam and a measurement beam by a beam splitter. After contacting the sample, the measurement beam is reflected and superimposed with the reference beam in an interferometer. A spectrometer records the resulting interference signal, which contains information about structures and their position. The corresponding software calculates this information. Information along the Z-axis at the measured points enables three-dimensional imaging across multiple measurements along the X- and Y-axes [27,28]. A GANYMEDE system with an LSM03-BB objective lens from Thorlabs (Gan210-SP4, Thorlabs, Dachau, Germany) was used for the recordings. The analysis programs were ThorImage version 5.1.1 and Fiji (ImageJ version 1.53/2.1.0) [27,29]. The data was analyzed according to Wagner and Horn [30].

#### 2.5.2 Biofilm quantification via crystal violet assay

To quantify biofilm formation of the deletion mutant compared to the wild type, an adapted Crystal Violet (CV) assay was performed in a 96-well plate format. The isolates were first grown overnight in LB medium, and the OD_600_ of the overnight cultures was measured. Cultures were then adjusted to an OD_600_ of 0.15 in 200 μL modified minimal medium (MM; DSMZ Medium 81, containing 2.5 mg ferric ammonium citrate instead of the standard 50 mg) supplemented with 20 mM fructose. To minimize evaporation, the outer wells of the plate were filled with uninoculated minimal medium, and the plate was covered with a lid. The cells were incubated at 30 °C for 72 h under static conditions.

After incubation, the OD_600_ of each well was measured using an Infinite Pro 200 spectrometer (Tecan, Switzerland) to assess total growth in the well. The medium was then removed, and the wells were washed three times with minimal medium to remove non-adherent cells. Adherent biomass was stained by adding 300 μL of 0.1% CV solution to each well, followed by incubation for 15 min. The CV solution was removed, and the bound dye was solubilized from the biofilm with 200 μL of 30% acetic acid for 10 min. The absorbance of the solubilized CV was then measured at 590 nm (A_590_) using the Infinite Pro 200 spectrometer.

Absolute CV staining was evaluated based on the A_590_ values. In addition, to account for differences in total biofilm formation between strains, a growth-normalized biofilm ratio was calculated for each replicate as A_590_/OD_600_. For relative comparison, this index was normalized to the mean value of the wild-type strain grown on the same 96-well plate. Thus, the normalized values represent relative, growth-normalized values rather than absolute biofilm biomass. The wild-type as well as the deletion strain were tested in nine replicate wells. Statistical analysis was performed on the replicate-level values using unpaired t-tests. The growth-normalized biofilm index is presented in Fig. 2 while measured OD_600_-and A_590_-values are presented in the supplemental material.

### 2.6 Statistical analysis

#### 2.6.1 Sequencing of the transposon library

Analysis of the sequencing data was done as described in Lapp et al. (2026) [20].

To identify enriched integrations, we normalized the number of integrations per position to the sequencing depth, yielding integrations per million (IPM). The corresponding genes were then annotated. For comparison with the initial library, we further normalized the total integrations per gene to the gene length, resulting in integrations per kilobase million (IPKM; Supplementary Material). Comparing these IPKM values with the initial library revealed no pronounced co-enrichment in specific genes. Furthermore, since integration positions in the initial library are inherently non-random, as described by Lapp et al. (2026) [20], we defined the most prominent hit genes independently of the library comparison.

#### 2.6.2 Biofilm development under fluidic conditions

Biofilm development curves were analyzed in R version 4.6.1 (“Happy Hop”; R Foundation for Statistical Computing) using RStudio version 2026.08.1 Build 195 (“Yellow Yarrow”) and the Growthcurver package version 0.3.1 by Sprouffske and Wagner (2016) [31]. Each biological replicate (n *=* 3 per strain) was fitted individually using the logistic growth model implemented in Growthcurver, yielding the rate parameter (*r*), modeled carrying-capacity parameter (*K*), inflection time (*t*_mid_), and area under the fitted logarithmic curve. The time required to reach 20% of the modeled maximum biofilm volume (*t*_20_) was derived from the fitted parameters. Model fit quality was assessed by calculating the coefficient of determination (R^2^) from observed and model-predicted biofilm volumes. R^2^ values ranged from 0.985 to 0.993 with a mean of 0.990 for B2043 and 0.992 for the wild-type.

In addition, the specific biofilm accumulation rate (μ_biofilm_) was calculated conventionally from the natural logarithm of the measured biofilm volume according to

(2) $$\left(\mu_{\left\{\text{biofilm}\right\}}=\frac{\left[\ln\left(X_{2}\right)-\ln\left(X_{1}\right)\right]}{t_{2}-t_{1}}\right)\text{.}$$

Equal 28-h intervals covering the corresponding major biofilm-accumulation phase were used for both strains (hours 32–60 for B2043 and hours 40–68 for the wild-type).

Differences between B2043 and the wild-type were assessed using two-sided Welch's two-sample *t*-tests. Data are reported as mean ± standard deviation (SD) of three biological replicates, while *p* < 0.05 was considered statistically significant.

## 3 Results

To identify key genes for steering biofilm formation in *C. necator*, a transposon-induced mutation library approach was chosen. After verification of the genome wide coverage of the mutation library, as described in detail in Lapp et al. (2026) [20], a biofilm formation-fitness experiment was conducted with the mutation harboring cells within a microfluidic biofilm cultivation platform. Cells were cultivated until a mature biofilm state was reached (168 h), after which the biomass was harvested and re-sequenced to assess which transposon-induced mutations granted a fitness benefit in biofilm formation and were enriched over the runtime of the experiment. To specify, biofilms were considered mature when surface-associated growth resulted in multilayered, three-dimensional structures within the cultivation channel, that had progressed considerably beyond the phases of initial surface attachment and early microcolony formation and entered a stationary biovolume state.

**Fig. 1:**
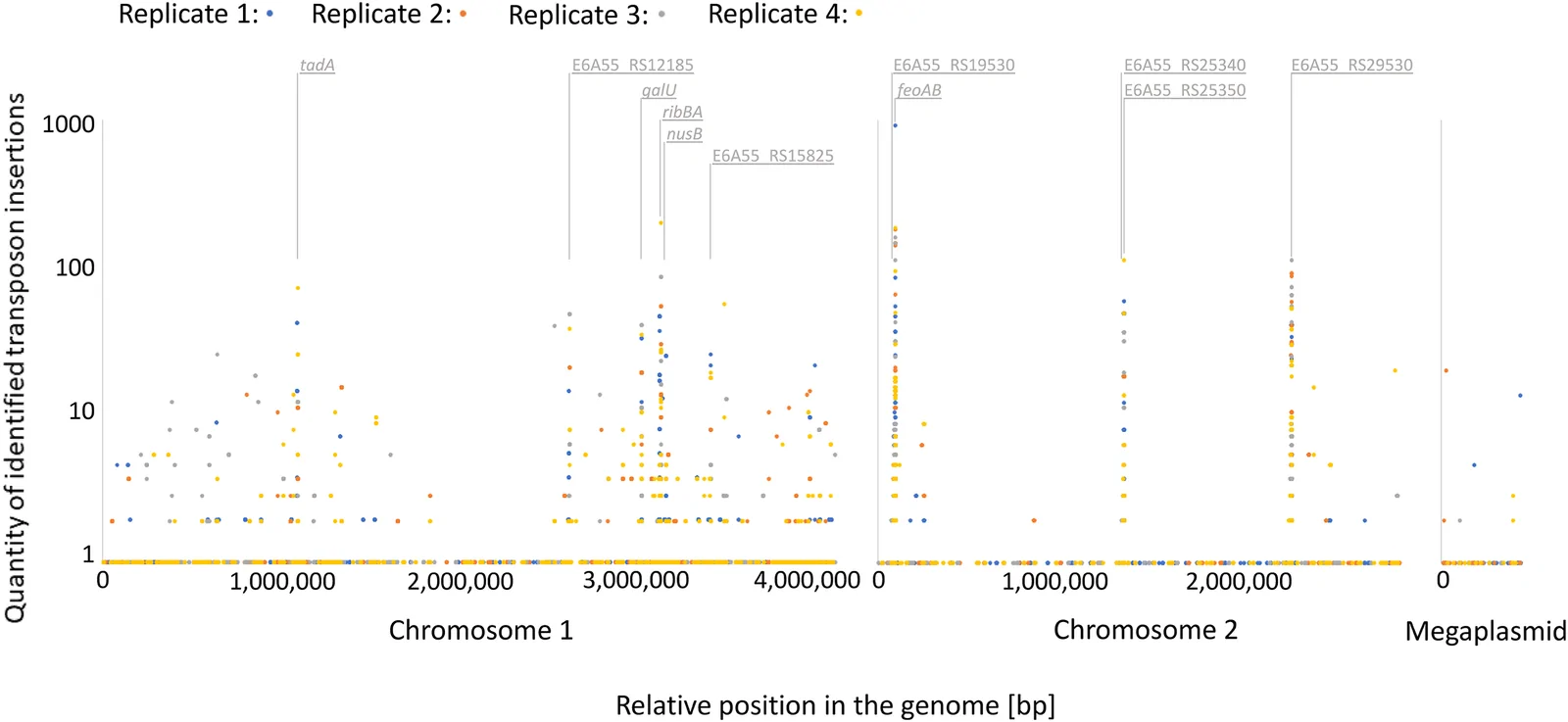
Distribution of the identified mutations in the genome. A mutation library was cultivated as a biofilm, resulting in the identification of mutations within the community of the four flow cells, which are represented by yellow, blue, orange, and grey. The quantity of identified mutations in the respective gene is displayed on the Y-axis, while the relative position of the gene in the genome of *C. necator*, which is sectioned in two chromosomes and one megaplasmid, is displayed on the X-axis in base pairs equivalent to locus area in Table 3. Chromosome 1, 2, and the megaplasmid are shown from left to right. The supplemental material presents a figure illustrating the distribution of the identified transposon insertion in the original mutation library. Genes indicated by name are listed in Table 3. (For interpretation of the references to colour in this figure legend, the reader is referred to the Web version of this article.)

The distribution of the identified transposon insertions in *C. necator* cells is shown as locations of the hit genes in relation to the genome in Fig. 1. The supplemental material shows a figure depicting the distribution of the identified transposon insertions in the original mutation library.

**Fig. 2:**
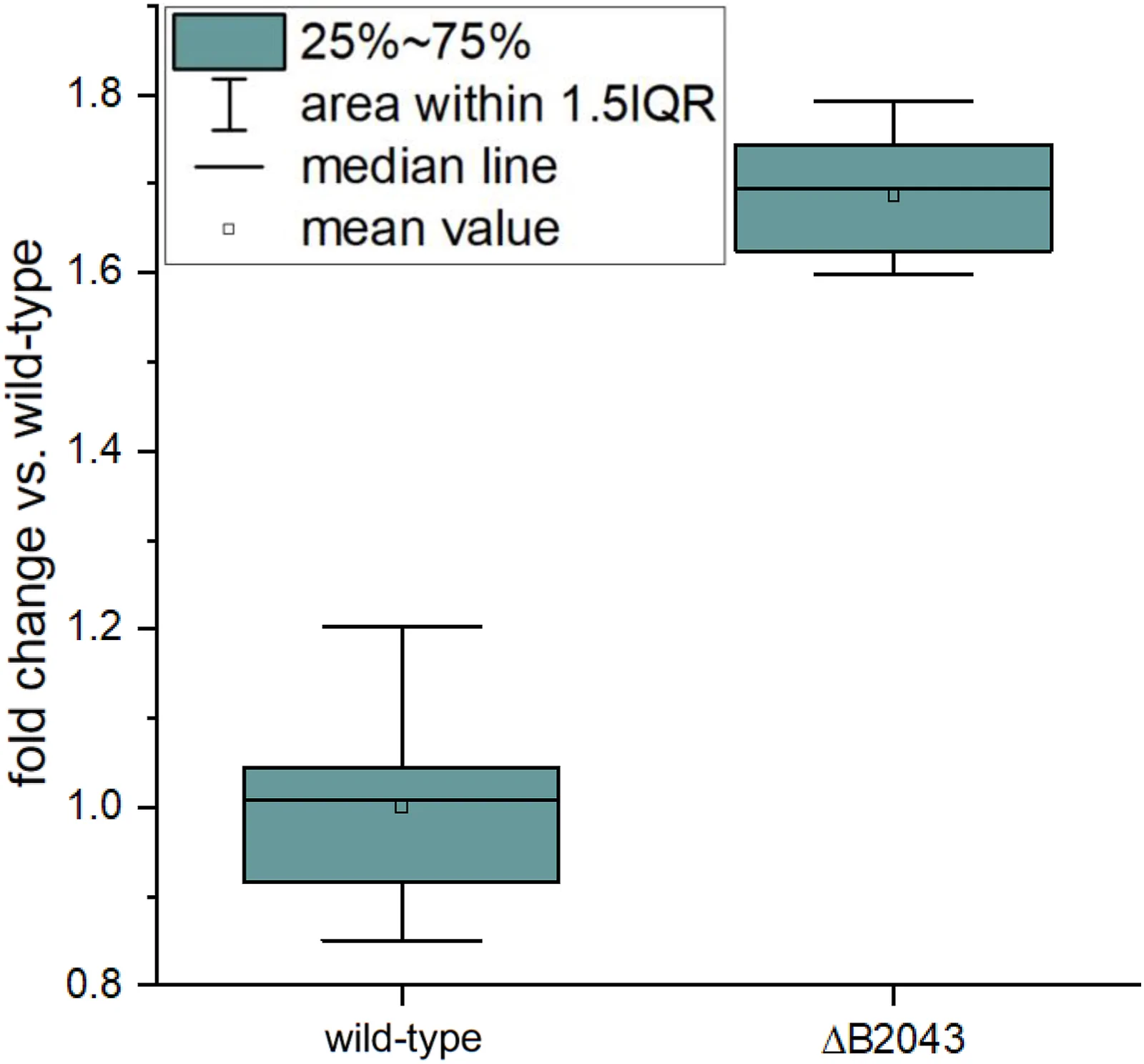
Biofilm growth under static, heterotrophic conditions shown as fold change compared to the parent strain. The plot shows the median (black line), upper and lower quartile as a box and whiskers indicating variability outside the upper and lower quartiles.

The resulting identified mutations were then subjected to analysis and prioritization, as detailed in the statistical analyses section of this work. Based on the IPM metric, we identified 12 candidate genes with the highest position-specific insertion rates (exceeding 2 IPM). These 12 genes also ranked among the top 30 genes with the highest IPKM values. Notably, the remaining 18 genes in this top-30 cohort are located in close physical proximity to the 12 primary candidates. Finally, a comprehensive literature review on these 12 genes was conducted to select a prime candidate for further investigation. Table 3 presents a list of the 12 identified genes, along with the median number of hits per gene, the maximum number of insertions at the same gene location, the gene locus area, and the gene function, as described in the National Center for Biotechnology Information (NCBI) database. The alternative gene identifiers, when available, are listed in the supplemental materials.

**Table 3:** Most hit genes identified in a biofilm formation fitness experiment with a transposon insertion library. Genes are presented with their respective gene identifiers, median of hits per gene, the maximum number of insertions at the same location within the genes, the locus area of the genes, and their function as described in the database of *NCBI*. Alternative gene IDs are listed in the supplemental material.

*Gene ID*	*Median hits per gene (IPKM)*	*MAX hits per location (max. IPM)*	*locus area*	*function (as described on NCBI)*	
*feoA*	245.5	142.9	98369.98683	Fe2+ transport protein FeoA [Inorganic ion transport and metabolism]	
E6A55_RS29530	67.6	17.3	2298052.2299380	Cyclic di-GMP metabolism protein, combines GGDEF and EAL domains with a 6TM membrane domain [Signal transduction mechanisms]	
*feoB*	62.6	27.9	96497.98353	Fe2+ transport protein FeoB [Inorganic ion transport and metabolism]	
*ribBA*	36.1	19.4	3077812.3078921	bifunctional 3,4-dihydroxy-2-butanone -4-phosphate synthase/GTP cyclohydrolase II	
*nusB*	26.0	12.5	3079487.3080005	transcription antitermination factor *nusB*	
E6A55_RS25350	27.0	12.6	1367230.1368135	DNA-binding transcriptional regulator, MurR/RpiR family, contains HTH and SIS domains [Transcription]; COG1737	
*galU*	13.9	8.8	2977625.2978515	UTP-glucose-1-phosphate uridylyltransferase [Cell wall/membrane/envelope biogenesis]; COG1210	
E6A55_RS12185	12.1	9.3	2577378.2578238	Metal-dependent hydrolase, endonuclease /exonuclease/phosphatase family [General function prediction only]; COG3568	
E6A55_RS19530	11.4	3.8	96165.96482	hypothetical protein; Family of unknown function (DUF6587); pfam20228	
E6A55_RS25340	7.3	2.6	1365310.1365702	RidA family protein; Enamine deaminase RidA, house cleaning of reactive enamine intermediates, YjgF/YER057c/UK114 family [Defense mechanisms]; COG0251	
E6A55_RS15825	6.8	3.4	3357202.3358245	type IV pilus twitching motility protein PilT; Type IV pilus assembly protein PilT, pilus retraction ATPase [Cell motility, Extracellular structures]; COG2805	
*tadA*	3.8	3.4	1074391.1076250	tRNA (Arg) A34 adenosine deaminase TadA; [Translation, ribosomal structure and biogenesis]; COG0590	

Of the here identified genes, several have literature-supported links to biofilm formation, which are discussed in further detail in the subsequent discussion section of this manuscript. E6A55_RS29530 (hereafter referred to as B2043 based on the alternative gene identifier shown in the supplemental material) was selected for further analysis based on existing literature support of the link to biofilm formation for this enzyme class.

### 3.1 Impact of B2043 under static biofilm conditions

To determine the contribution of B2043 to biofilm formation, the marker-less deletion mutant was first analyzed under static conditions using a CV assay after 72 h of cultivation and compared to the parent strain. The ΔB2043 mutant showed increased CV-stained biofilm biomass after 72 h. In addition, the growth-normalized biofilm index A_590_/OD_600_ was 1.69 ± 0.18-fold higher than in the wild type as shown in Fig. 2, indicating that deletion of B2043 increased biofilm formation. The difference proved to be highly significant (p = 5.16 × 10^−15^, unpaired *t*-test) indicating a robust phenotype (p = 2.63 × 10^−6^ and p = 7.08 × 10^−14^ for raw data shown in supplemental material).

### 3.2 Impact of B2043 under flow-through conditions

**Fig. 3:**
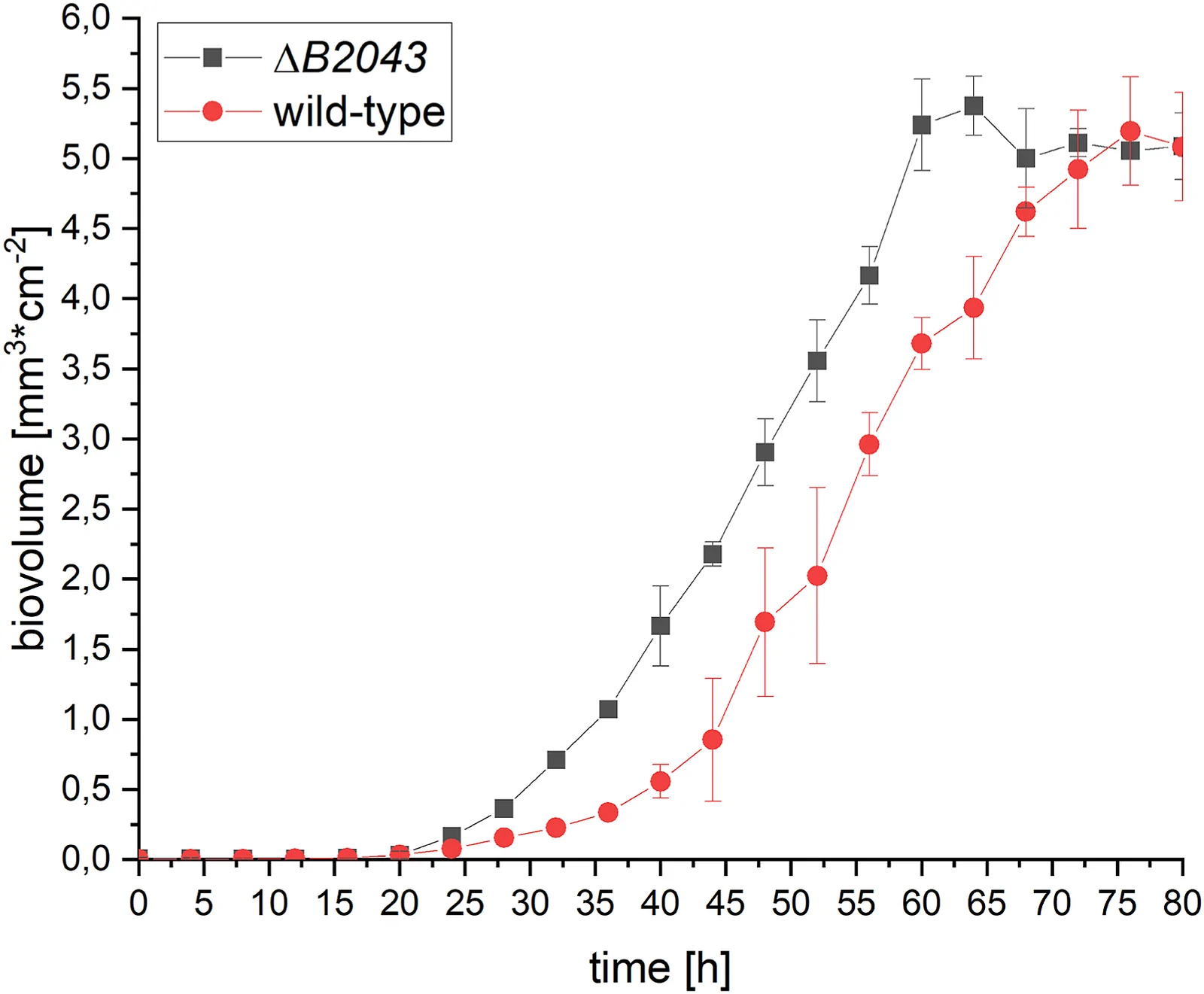
Biovolume of heterotrophic biofilm grown under flow-through conditions of the B2043-mutant strain shown in grey compared to the wild-type strain in red. The mutant strain entered the exponential growth phase 10 h earlier and reached a plateau phase 16 h earlier then the wild-type strain, showing a shorter attachment and lag phase. Error bars represent the calculated standard deviation per oct imaging point between the respective triplicates. (For interpretation of the references to colour in this figure legend, the reader is referred to the Web version of this article.)

In order to extend the analysis beyond static biomass accumulation, biofilm development was examined under flow-through conditions. These conditions allow for chemostat conditions, with continuous nutrient supply, and a more detailed analysis of biofilm architecture. In this configuration, the process of biofilm formation and maturation was observed over time through the use of optical coherence tomography (OCT), a technique that allows for the quantitative evaluation of both the height of the biofilm and its three-dimensional structure in comparison to the wild-type strain. Measured biovolume of the triplicate flow cells is illustrated in Fig. 3 over the time period of the experiment.

Both triplicates exhibited initial growth after an extended lag phase, which occurred at approximately 20 h. The mutant strain rapidly entered the exponential growth phase after 32 h, reaching a plateau phase at approximately 5.24 mm^3^ per cm^2^ at 60 h. In contrast, the wild-type replicates entered the exponential phase after a slower initial period, at around 40 h, and reached a comparable plateau phase of around 5.2 mm^3^ per cm^2^ after 68 h, with an 8-h delay.

Quantitative analysis of the biofilm development curves using a growth model revealed comparable biofilm accumulation rates (*r*) for B2043 and the wild-type strain (0.150 ± 0.006 h^−1^ and 0.146 ± 0.028 h^−1^, respectively; Welch's *t*-test, *p* = 0.837). Likewise, the carrying-capacity parameter (k), which represents the total asymptotic biofilm volume, did not differ significantly between B2043 and the wild-type (5.30 ± 0.12 and 5.33 ± 0.55, respectively; *p* = 0.929). Consistent with the biofilm accumulation rate parameter, the conventionally calculated specific biofilm accumulation rate (μ_biofilm_) was comparable for B2043 and the wild-type (0.076 ± 0.006 h^−1^ and 0.071 ± 0.001 h^−1^, respectively). Despite the comparable accumulation rates and carrying capacities, the overall area under the biofilm development curve (AUC) was significantly larger for B2043 than for the wild-type strain (180.3 ± 3.6 vs. 135.3 ± 4.2, respectively; *p* = 0.00017) indicating a pronounced temporal shift in biofilm development. The inflection point of the fitted curve (*t*_mid_), corresponding to the time at which 50% of the biovolume was reached, occurred approximately 8.7 h earlier in B2043 than in the wild-type (46.0 ± 1.2 h vs. 54.7 ± 3.0 h; *p* = 0.025).

Inspection of the biofilm development curves suggested that both strains initially exhibited a comparable early phase up to 20 h, followed by an earlier onset of biofilm accumulation in B2043, whereas subsequent biofilm development appeared similar once the wild-type had reached the corresponding developmental stage. To quantify this temporal shift, the time required to reach 20% of the modeled carrying capacity (*t*_20_) was calculated. B2043 reached this threshold approximately 8.2 h earlier than the wild-type (36.7 ± 0.9 h vs. 45.0 ± 3.0 h; *p* = 0.032). Taken together, these analyses indicate that the increased overall biofilm accumulation observed for B2043 is primarily associated with an approximately 8–9 h faster transition from the attachment phase to a pronounced biofilm accumulation and therefore the biofilm maturation phase, rather than with an increased accumulation rate or a higher final biofilm forming capacity.

**Fig. 4:**
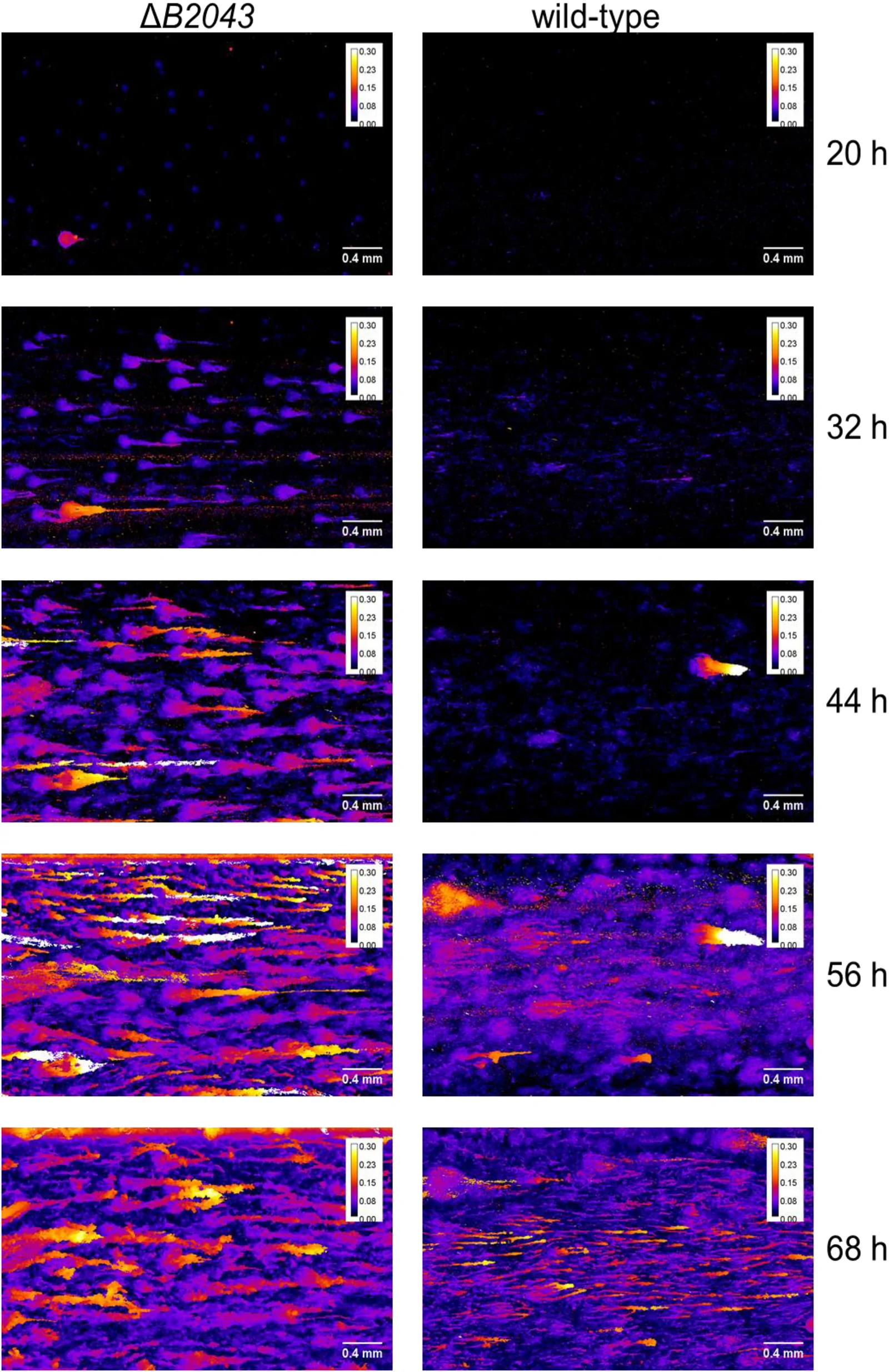
Height maps of the biofilms under heterotrophic flow-through conditions made with OCT imaging. Presented is an image for every 12 h of the experiment within the late attachment phase and partially of the exponential growth phase. Images on the left show biofilm formation by the ΔB2043 mutant strain, while images on the right represent images of the biofilm formation by the wild-type strain.

To further examine the initial attachment and the morphology of the biofilm in the flow-through experiment, OCT images were examined in over the course of the experiment, as illustrated in Fig. 4.

Height maps of the same positions in the flow cells are shown every 12 h in the late settling and exponential phase, between 20 and 68 h. After 20 h, the B2043-mutant strain shows colonies. In contrast to the wild-type strain, these colonies stay in their round shape in a very distinct manner, growing mainly in height into tower-like structures, until the constant shear force led to streamer-like formations between 32 and 44 h. In contrast, the wild-type strain exhibits a more diffuse colonization of the substratum, with smaller colonies but a more uniform layer of biofilm, showing small streamers and tower-like structures only after 56 to 68 h of the experiment.

## 4 Discussion

### 4.1 Identified genes linked to biofilm formation

Of the genes identified in this study via transposon insertion, several have literature-supported links to biofilm formation, although the strength and direction of these associations differ between genes and organisms.

For *feoA*, a direct connection to biofilm-associated phenotypes has been reported. In *Acinetobacter baumannii*, *feoA* was required for growth, cellular adhesion, and biofilm formation, and the respective mutant showed reduced colonization and virulence as well as increased susceptibility to oxidative stress [32]. In addition, Feo-mediated ferrous iron uptake appears particularly relevant under the low-oxygen conditions that prevail in biofilms [33]. In *P. aeruginosa* biofilms, such as those found in cystic fibrosis lungs, ferrous iron is more prevalent than ferric iron, and the Feo system is used for iron acquisition under these conditions [[34], [35], [36], [37]]. For *feoB*, the major component of the Feo system, the evidence is similarly direct and even more extensive. In *P. aeruginosa*, FeoB contributes to survival in the anaerobic biofilm environment and is involved in Fe^2+^-mediated biofilm formation [35,38,39]. Additionally, in *Aeromonas veronii*, deletion of *feoB* significantly reduced biofilm formation, which was accompanied by impaired motility, adhesion, and invasion, consistent with a broader role of intracellular iron homeostasis in biofilm development [40]. The system has exclusively been shown to positively influence biofilm formation in other organisms. Consequently, it is plausible that the system in *C. necator* differs from that in other organisms, exerting a detrimental influence on biofilm formation or the phenomenon may be attributable to a yet unidentified background mechanism and secondary effect. Alternatively, it is possible that the organism profits more from energy conservation than from a functional Feo-system in the used cultivation platform. According to the KEGG database, 21 iron transporters have been identified in *C. necator*. However, only two of these are annotated for ferrous iron: *feoA* and *feoB*. This observation suggests that in the used chemostat-like biofilm cultivation system, the prevalence of ferrous iron through oxygen gradients may not be as prevalent as would be expected. Consequently, the utilization of transporters would not be necessary, and their presence would constitute a potential energy expenditure.

Although E6A55_RS29530 is annotated in NCBI as encoding a GGDEF/EAL dual-domain protein, closer inspection revealed a non-canonical GSDEF/EAL domain architecture. This distinction is relevant as the canonical GGDEF motif is associated with diguanylate cyclase activity, whereas substitutions within this motif often indicate degenerate GGDEF-like domains with reduced or absent c-di-GMP synthesis activity. E6A55_RS29530 should therefore not be interpreted as a predicted bifunctional diguanylate cyclase/phosphodiesterase enzyme, but rather as a putative c-di-GMP-related signaling protein carrying a degenerate GGDEF-like domain and an EAL domain. GGDEF- and EAL-domain proteins are central regulators of c-di-GMP metabolism and are strongly linked to biofilm regulation in many bacteria. In general, high intracellular c-di-GMP levels promote sessile growth and biofilm formation, whereas low levels favor motility [4,41]. Several EAL-containing proteins have been shown to act as negative regulators of biofilm formation, including BifA in *P. aeruginosa*, CdgJ in *Vibrio cholerae*, and GepA in *Vibrio parahaemolyticus* [[42], [43], [44], [45]]. Importantly, GGDEF-like domains can retain regulatory functions even when they lack diguanylate cyclase activity. For example, the GSDEF/EAL protein SesB from *Thermosynechococcus vulcanus* lacks detectable diguanylate cyclase activity but exhibits light-regulated c-di-GMP phosphodiesterase activity [46]. Similarly, the degenerate GEDEF/EAL protein PdeA from *Caulobacter crescentus* lacks diguanylate cyclase activity, but its GGDEF-like domain binds GTP and allosterically stimulates the EAL phosphodiesterase domain [47]. By analogy, the GSDEF domain of E6A55_RS29530 may act as a regulatory GGDEF-like module, while the EAL domain represents the more plausible catalytic domain. In this context, the directly adjacent gene E6A55_RS29535 encodes a predicted methyl-accepting chemotaxis protein (MCP), suggesting a possible second sensory-regulatory context. MCP-associated c-di-GMP signaling modules are known from other bacteria, most prominently the Wsp system of *P. aeruginosa*, in which WspA contributes to surface sensing and activates the GGDEF-domain diguanylate cyclase WspR, thereby promoting biofilm matrix production [41,[48], [49], [50]]. The genomic proximity of E6A55_RS29530 and E6A55_RS29535 may therefore indicate a Wsp-like sensory-regulatory module in *C. necator*, although this remains hypothetical and requires experimental validation.

*ribBA* can only be linked indirectly to biofilm formation and the link remains speculative. In *Sinorhizobium meliloti*, *ribBA* encodes a bifunctional enzyme involved in riboflavin biosynthesis, and its deletion impaired flavin secretion, root colonization, and competitiveness for nodulation [51]. In *Shewanella*-species, secreted flavins, including riboflavin, mediate extracellular electron transfer in biofilms and biofilm formation, indicating that flavin biosynthesis and secretion can support biofilm-associated physiology, although a direct role of *ribBA* in biofilm formation itself was not yet demonstrated [[52], [53], [54]].

For *nusB*, no direct connection to biofilm formation could be identified either. NusB is a conserved transcription antitermination factor required for rRNA transcription antitermination in *E. coli* rRNA, which refers to the suppression of premature transcription termination during rRNA operon expressions [55]. Although RNA-based antitermination has been linked in other bacterial systems to the regulation of exopolysaccharide operons associated with biofilm formation, most notably through the EAR element in *Bacillales* [56], a direct role of NusB in biofilm regulation has not been demonstrated yet. Any effect could therefore be linked to its broader role in transcription of rRNA.

For E6A55_RS25350, a predicted MurR/RpiR-family transcriptional regulator, no direct link to biofilm formation could be identified as well. Members of this family contain an N-terminal HTH DNA-binding domain and a C-terminal SIS domain and are mainly associated with sugar-phosphate metabolism, while MurR itself acts as a repressor of murein catabolism in *E. coli* [57]. A possible contribution to biofilm-related phenotypes would therefore remain indirect, for example through effects on cell wall turnover or metabolism.

Among the genes considered here, *galU* has one of the strongest and most broadly supported associations with biofilm formation. *galU* encodes for an UDP-glucose pyrophosphorylase, which is required for UDP-glucose production and thereby contributes to lipopolysaccharide, capsular polysaccharide, and exopolysaccharide biosynthesis. These surface and matrix components are directly relevant to biofilm formation [58]. In *Salmonella typhimurium*, *galU* mutation reduced biofilm formation, autoaggregation, and motility, while increasing sensitivity to serum and antibiotics [59]. Literature further indicates effects of *galU* on biofilm formation and colonization in *Streptococcus pneumoniae* and in *V. cholerae* a *galU* mutant was reported to be biofilm negative [60,61]. However, the effect is not universally conserved, as in *Pseudomonas nitroreducens* TX1 a *galU* mutant displayed increased biofilm formation and elevated antibiotic resistance [62]. This implicates that the contribution of *galU* to biofilm phenotypes may depend on the organism. As revealed in this study for *C. necator*, the data strongly suggests a mutation of *galU* to be biofilm promoting.

No direct evidence linking E6A55_RS12185, which is annotated as a metal-dependent hydrolase of the endonuclease/exonuclease/phosphatase family, to biofilm formation could be identified. Similarly, no literature could be found for E6A55_RS19530, as neither its function nor its family are known.

The RidA protein family is conserved across all domains of life and functions as a metabolic housekeeping enzyme that detoxifies reactive enamine/imine intermediates, principally 2-aminoacrylate (2AA), which are generated as by-products of pyridoxal 5′-phosphate (PLP)-dependent reactions. In the bacterium *P. aeruginosa* PAO1, a transposon insertion mutant lacking the PA5339 protein (subsequently designated RidA) displayed significant defects in growth, motility, and biofilm formation. These defects could all be mechanistically linked to the intracellular accumulation of 2AA. The PA5339 protein was confirmed as a functional 2AA deaminase in vitro, restoring metabolic balance to a *Salmonella enterica ridA* mutant in vivo and demonstrating functional conservation [63]. The data indicates that loss of RidA function leads to broad metabolic disruption via the inactivation of multiple PLP-dependent enzymes, resulting in defective biofilm formation [63,64]. The contrary effect indicated in this work for E6A55_RS25340 is not supported by literature and should be further investigated for *C. necator*.

E6A55_RS15825 is described as the type IV pilus twitching motility protein PilT and therefore the retraction ATPase of the type IV pilus machinery. It is responsible for pilus depolymerisation and generating mechanical force during twitching motility. Deletion of PilT results in a hyperpiliated, non-twitching phenotype due to an inability to retract assembled pili. Under static growth conditions, hyperpiliated, non-twitching Δ*pilT* mutants of *P*. *aeruginosa* formed dense biofilms, demonstrating that surface adhesion mediated by accumulated pili rather than twitching motility per se is the primary determinant of biofilm initiation. Under flow conditions, the Δ*pilT* mutant additionally formed mushroom-like structures that were larger than those of the wild-type, indicating that PilT-mediated retraction modulates biofilm architecture, likely through the regulation of surface detachment [65]. Similar findings have been reported in *Geobacter sulfurreducens*, where inactivation of the cognate PilT4 ATPase produced a hyperpiliated phenotype associated with increased cell-to-surface adhesion and denser biofilm formation under static conditions. This phenotype was completely reversed through genetic complementation [66]. Therefore, the relationship between *pilT* and biofilm formation is positive with respect to biofilm promotion upon gene deletion: loss of PilT function leads to pilus accumulation on the cell surface, which increases initial adhesion and results in enhanced biofilm initiation and development as is indicated through this work for *C. necator*.

The *tadA* gene encodes the prokaryotic tRNA-specific adenosine deaminase that is responsible for A-to-I editing in prokaryotes. This gene has been linked to biofilm formation due to its role in mRNA editing under conditions of oxidative stress. In *Xanthomonas oryzae* pv. *oryzicola*, TadA has been shown to catalyse a non-synonymous A-to-I editing event (S128P) in the *fliC* mRNA, which encodes the flagellar filament protein. This editing event alters flagellar filament structure, increasing surface motility and enhancing biofilm formation, as quantified by confocal laser scanning microscopy. Deletion of *tadA* abolished this editing and was associated with reduced virulence and diminished biofilm-associated phenotypes. Conversely, overexpression of *tadA* increased editing levels and biofilm formation. The gene cluster for siderophore biosynthesis and Fe^3+^ uptake was co-upregulated in the edited background, suggesting that TadA-mediated RNA editing integrates oxidative stress responses with biofilm-promoting behaviors. These findings suggest a positive correlation between TadA activity and biofilm formation, indicating that loss of *tadA* function is associated with reduced biofilm capacity in this organism [67] contrary to what is implied in this work for *C. necator*.

Among the identified and described candidates, the genes of the Feo-system (E6A55_19525 and E6A55_19520) exhibited the highest rate of insertions. However, given the elusiveness of the connection of the system to biofilm regulation and the contrary direction of regulation indicated in literature, secondary effects cannot be ruled out. Therefore, B2043 (name based on the alternative gene identifier shown in the supplemental material), encoding the GSDEF/EAL dual-domain protein, was selected for further analysis, as it demonstrates the strongest and most direct link to biofilm regulation among the genes identified here. The substantial support from existing literature and the second highest number of insertions identified in the biofilm fitness experiment make it the ideal candidate for preliminary examination of the identified genes in this work.

### 4.2 Impact of B2043 under static biofilm conditions

The data obtained from the CV assay corroborates the initial findings of the transposon screen, thereby demonstrating that B2043 acts as a negative regulator of biofilm accumulation under static growth conditions in *C. necator*. In light of the aforementioned literature, the data corroborates that the EAL-domain is the catalytic active domain while the GSDEF domain is catalytic non-functional.

### 4.3 Impact of B2043 under flow-through conditions

To extend the analysis beyond static biomass accumulation, the development of biofilms was examined under flow-through conditions. As illustrated in Fig. 3, the biovolume of triplicate flow cells was measured via OCT.

The findings of the flow-through experiment indicate that the mutant strain may be distinguished by a more pronounced attachment, which accelerates initial growth as a biofilm under flow-through conditions. This enhanced attachment of the cells might also provide a rationale for the observed difference in biovolume in the CV assay. An initial attachment of cells that is pronounced would result in a greater amount of the limited carbon being used for biofilm volume. Concurrently, an increase in planktonic cells would enhance competition for the carbon source. The assay is normalized to the total OD of all cells. In the wild-type, a relatively greater number of planktonic cells were present as evident by the greater measured OD-values, resulting in a smaller volume of biofilm. In contrast to the static biofilm assay, the flow cell experiments demonstrated no higher yield of maximum biofilm volume compared to the wild-type strain. These findings support the hypothesis of a pronounced attachment rather than a faster biofilm growth corroborated by the statistical analysis of the experiment, which showed only a significant difference in hours 20 to 45 between the strains.

Height map images in Fig. 4 provide further evidence that supports the hypothesis that the cells exhibit an enhanced attachment to the substratum, as well as cell-to-cell adhesion within the biofilm. Furthermore, the observed distinctive morphology may offer an additional competitive advantage to the mutant strain in terms of static biofilm growth. The robust, tower-like structures maintain their integrity throughout the experiment, even under flow-through conditions, which could facilitate unobstructed nutrient diffusion to the biofilm base. In contrast, a more uniform growth pattern might impede nutrient diffusion and generate gradients, as is often observed in biofilm systems [68].

### 4.4 Comparison with deletion of other EAL-domain-containing genes

The phenotype observed for the deletion mutant is consistent with previous reports on EAL-domain-containing proteins acting as negative regulators of biofilm formation. In the present study, the deletion of the EAL-domain-containing gene resulted in enhanced attachment under flow-through conditions, a distinct biofilm morphology, increased cell aggregation, and increased biovolume in the static CV assay. Specifically, the mutant demonstrated approximately 1.7-fold higher biofilm formation in the static assay and reached the exponential growth phase faster as well as a biofilm volume comparable to the maximum volume of the wild-type approximately 16 h earlier in the flow-through system. It should be noted that analogous phenotypes have been described for other c-di-GMP phosphodiesterases. In *P. aeruginosa*, the deletion of *bifA*, which encodes a GGDEF-EAL protein with phosphodiesterase activity, increased biofilm formation by approximately 5.5-fold, measured by CV, and was associated with elevated intracellular c-di-GMP levels [42]. Likewise, loss of the EAL-domain containing WspF regulator in the Wsp chemosensory system increased c-di-GMP levels and accelerated surface attachment, resulting in approximately 6-fold enhanced attachment [41]. Comparable effects were also reported for the EAL-domain phosphodiesterase CdpA in *Burkholderia pseudomallei*, where the *cdpA* mutant showed increased c-di-GMP levels, enhanced autoaggregation, increased cell-to-cell adhesion, and approximately 3.7-fold higher biofilm formation in a CV assay [69]. The visible clumping described for the *cdpA* mutant in static liquid cultures is particularly similar to the increased aggregation observed in the precultures of the present study (data not shown) and to the attachment, as well as resulting structures, detected after 32 h in the OCT flow-through data. Additional examples include *cdgJ* in *V. cholerae*, where deletion increased biofilm formation by approximately 1.8-fold despite no clear change in bulk c-di-GMP levels [44], and *gepA* in *V. parahaemolyticus*, where deletion of the EAL-domain increased intracellular c-di-GMP and enhanced biofilm formation by approximately 6-fold [45]. In *Yersinia pestis*, the EAL-domain protein HmsP has also been described as a phosphodiesterase required for negative regulation of biofilm formation. The loss of HmsP activity has been shown to result in a hyper-biofilm phenotype, marked by increased cell aggregation in Congo Red-based assays [70].

Taken together, these studies support the interpretation that deletion of the GSDEF-EAL-domain-containing gene in the B2043 mutant shifts the cells toward a more sessile, adhesive, and aggregated phenotype, potentially through increased c-di-GMP levels. However, the comparatively moderate increase in total static biofilm formation and the mainly accelerated development in the flow-through system suggest that the deletion primarily affects the initial attachment, aggregation and structural development of biofilm formation rather than causing a strongly enhanced final biofilm capacity. Nevertheless, mass transfer limitations within the system will probably preclude a comparison of final biovolume in the flow-through experiment, as physical gradients will affect both biofilm triplicates equally at a comparable amount of biovolume [[71], [72], [73]]. Variations in shear force, resulting from pressure changes caused by the medium, attributable to volumetric constraints during the biofilm's growth phase within the cultivation channel, will ultimately influence the timing of arising mass transfer limitations. However, it is likely unable to reverse these limitations [74]. Additionally, the same shear force change will constrain biovolume eventually. Consequently, a definitive conclusion regarding the impact of B2043 on biovolume remains elusive.

## 5 Conclusion

This study identifies B2043 as a regulator of biofilm formation in *C. necator*. The deletion of B2043 led to several notable changes, including faster settling, enhanced aggregation, altered biofilm morphology, and increased biofilm formation under static conditions. In a flow-through system, the mutant reached a biofilm volume comparable to the maximum wild-type biovolume and entered the exponential growth phase approximately 16 h earlier, while exhibiting tower-like structures, indicating that B2043 primarily affects the attachment phase and, through enhanced attachment and aggregation, also structural development of biofilm formation. Based on its GSDEF-EAL-domain and the observed phenotype, B2043 functions as a c-di-GMP phosphodiesterase and acts as a negative regulator of a sessile, adhesive lifestyle. This finding aligns with the observations made in other c-di-GMP regulatory systems, such as the Wsp system in *P. aeruginosa*, where the deletion of *wspF* led to a significant increase in c-di-GMP levels, a faster rate of attachment, and a notable change in the expression of at least 560 genes. This highlights the extensive regulatory influence that such systems can exert [41]. Consequently, such regulators hold considerable promise for industrial applications, as evidenced by the potential of c-di-GMP regulators to address the ongoing challenges posed by the absence of cleaning protocols for biofilm reactors and surfaces subject to biofouling [4,75]. The potential of c-di-GMP phosphodiesterases as molecular switches, capable of utilizing natural biofilm dispersion against biofouling in industrial reactors or, in biofilm-reactors, as a cleaning procedure, renders B2043 a promising candidate for an overexpression study in the future, to further the applicability of productive biofilm systems.

To our knowledge, this study is the first to provide experimental evidence that c-di-GMP-dependent regulation is involved in biofilm formation in *C. necator* H16. To date, the evidence for a direct connection between c-di-GMP and biofilm formation within this genus is primarily supported for the closely related species *C. metallidurans*. In this species, c-di-GMP levels have been associated before with EPS production and biofilm development [76]. The establishment of a c-di-GMP-based regulated biofilm formation provides a foundation for future research. A more direct approach can now be adapted to elucidate the mechanisms underlying biofilm formation in *C. necator*, as, for example, has been previously employed by Li et al. (2026), who specifically targeted enzymes involved in c-di-GMP regulation to identify diguanylate cyclases that regulate biofilm formation and antibiotic resistance in *A. baumanii* [77].

The transposon-based screening approach was highly valuable, as selection was driven by the biofilm formation pressure through the cultivation conditions, rather than by prior assumptions about candidate genes. This resulted in a set of putative biofilm-regulatory loci, of which the first selected candidate, B2043, was experimentally confirmed as relevant for biofilm formation and regulation, especially in the attachment phase. The remaining candidates therefore represent promising targets for future studies and may reveal additional regulatory pathways controlling biofilm development in *C. necator*. Further characterization of B2043, and the additional candidates identified in this screen, will help clarify how *C. necator* coordinates attachment, aggregation, biofilm architecture, and dispersal. Especially the Feo-system seems of high interest in *C. necator,* as indicated by the extremely high prevalence of insertions in the biofilm formation-fitness experiment. Additionally, 7 of the 12 identified candidate genes listed in this work have a direct link to biofilm formation in other organisms as supported by literature. Therefore, this work provides a template for further investigation of biofilm formation or genetic modifications to adjust *C. necator* in regard to productive biofilm systems, as they prove to be of high interest to tackle present challenges of biotechnology [4].

## CRediT authorship contribution statement

**Janek René Weiler:** Writing – review & editing, Writing – original draft, Validation, Methodology, Investigation, Formal analysis, Data curation. **Christian Jonas Lapp:** Writing – review & editing, Validation, Software, Methodology, Formal analysis. **Johannes Gescher:** Writing – review & editing, Supervision, Resources, Funding acquisition, Conceptualization. **Miriam Edel:** Writing – review & editing, Visualization, Project administration, Funding acquisition, Formal analysis, Data curation, Conceptualization.

## Declaration of competing interest

The authors declare that they have no competing interests.

## Data Availability

All data collected and reported in this study have been made available under copyright at TUHH Open19 Research (TORE): https://doi.org/10.15480/882.17412 NCBICurpiavidus necator transposon library (Original data)
