# Supplementary material for: Identification of genetic determinants that promote biofilm growth under heterotrophic conditions in Cupriavidus necator using transposon enrichment
Source: Biofilm. 2026 Sep 1;12:100395. doi: 10.1016/j.bioflm.2026.100395 (PMC13571940; doi:10.1016/j.bioflm.2026.100395)
Supplement: Supplementary file 1 [file mmc1.docx]

**Supplemental material**

S1: Alternative gene IDs for the identified candidate genes shown in Table 3.

| *Gene ID* | *alt. ID 1* | *alt. ID 2* |
| --- | --- | --- |
| *feoA* | E6A55_19525 | h16_B0085 |
| E6A55_RS29530 | E6A55_29510 | h16_B2043 |
| *feoB* | E6A55_19520 | h16_B0084 |
| *ribBA* | E6A55_14560 |  |
| *nusB* | E6A55_14570 | h16_A2856 |
| E6A55_RS25350 | E6A55_25330 | h16_B1210 |
| *galU* | E6A55_14050 | h16_A2752 |
| E6A55_RS19530 | E6A55_19515 |  |
| E6A55_RS12185 | E6A55_12175 | h16_A2371 |
| *tadA* | E6A55_04980 |  |
| E6A55_RS25340 | E6A55_25320 |  |
| E6A55_RS15825 | E6A55_15815 |  |

S2: Distribution of the identified mutations in the genome of *C. necator* cells of a Tn5-insertion library. The quantity of identified mutations in the respective gene is displayed on the Y-axis, while the relative position of the gene in the genome of *C. necator*, which is sectioned in two chromosomes and one megaplasmid, is displayed on the X‑axis in base pairs equivalent to locus area in Table 3. Chromosome 1, 2, and the megaplasmid are shown from left to right.

S3: OD_600_ and A_590_ of wildtype and *ΔB2043 used for biofilm quantification in crystal violet assay*.

| *Strain/replicate* | *A_590_* | *OD_600_* |
| --- | --- | --- |
| *WT/1* | 1.0066 | 1.372 |
| WT/2 | 1.1906 | 1.3813 |
| *WT/3* | 1.434 | 1.3834 |
| *WT/4* | 1.2347 | 1.3864 |
| *WT/5* | 1.0888 | 1.3798 |
| WT/6 | 1.1025 | 1.4248 |
| *WT/7* | 1.2044 | 1.4078 |
| WT/8 | 1.2989 | 1.379 |
| WT/9 | 1.2258 | 1.3985 |
| Mean | **1.199525** | **1.391858** |
| SD | **0.111641245** | **0.016013** |
| *ΔB2043/1* | 1.4445 | 1.0458 |
| *ΔB2043/2* | 1.7141 | 1.1094 |
| *ΔB2043/3* | 1.6926 | 1.1147 |
| *ΔB2043/4* | 1.6184 | 1.1324 |
| *ΔB2043/5* | 1.5606 | 1.075 |
| *ΔB2043/6* | 1.5385 | 1.1053 |
| *ΔB2043/7* | 1.5298 | 1.0861 |
| *ΔB2043/8* | 1.6258 | 1.1025 |
| *ΔB2043/9* | 1.4957 | 1.0863 |
| Mean | **1.596858333** | **1.097458** |
| SD | **0.085718572** | **0.023585** |
